# Copper Oxide Nanoparticles Alter Serum Biochemical Indices, Induce Histopathological Alterations, and Modulate Transcription of Cytokines, *HSP70*, and Oxidative Stress Genes in *Oreochromis niloticus*

**DOI:** 10.3390/ani11030652

**Published:** 2021-03-01

**Authors:** Hany M. R. Abdel-Latif, Mahmoud A. O. Dawood, Samy F. Mahmoud, Mustafa Shukry, Ahmed E. Noreldin, Hanan A. Ghetas, Mohamed A. Khallaf

**Affiliations:** 1 Department of Poultry and Fish Diseases, Faculty of Veterinary Medicine, Alexandria University, Alexandria 21544, Egypt; 2 Department of Animal Production, Faculty of Agriculture, Kafrelsheikh University, Kafrelsheikh 33516, Egypt; mahmoud.dawood@agri.kfs.edu.eg; 3 Department of Biotechnology, College of Science, Taif University, P.O. Box 11099, Taif 21944, Saudi Arabia; s.farouk@tu.edu.sa; 4 Department of Physiology, Faculty of Veterinary Medicine, Kafrelsheikh University, Kafrelsheikh 33516, Egypt; mostafa.ataa@vet.kfs.edu.eg; 5 Histology and Cytology Department, Faculty of Veterinary Medicine, Damanhour University, Damanhour 22511, Egypt; ahmed.elsayed@damanhour.edu.eg; 6 Department of Aquatic Animal Medicine and Management, Faculty of Veterinary Medicine, University of Sadat City, Sadat City 32897, Egypt; hanan.ghetas@vet.usc.edu.eg (H.A.G.); mohamed.khallaf@vet.usc.edu.eg (M.A.K.)

**Keywords:** Nile tilapia, CuONPs, histopathology, gene transcription, toxicity

## Abstract

**Simple Summary:**

Copper oxide nanoparticles (CuONPs) are increasingly manufactured because of their wide range of biomedical uses and industrial applications. Nonetheless, their release into the aquatic ecosystems is predictably increased, which will sequentially induce serious toxicological influences on the exposed aquatic biota. Several research studies have been published on CuONPs toxicity in fish; however, the mechanisms of their toxicity at the molecular levels in Nile tilapia (*Oreochromis niloticus*) are not completely described. The current study investigated the influences of sub-lethal CuONPs levels on serum biochemical indices, histopathological alterations, and transcriptomic responses in the hepatic and gill tissues of Nile tilapia juveniles.

**Abstract:**

In the present study, fish were exposed to sub-lethal doses of CuONPs (68.92 ± 3.49 nm) (10 mg/L, 20 mg/L, and 50 mg/L) for a long exposure period (25 days). Compared to the control group (0.0 mg/L CuONPs), a significant dose-dependent elevation in blood urea and creatinine values, serum alanine transaminase, aspartate transaminase, and alkaline phosphatase enzyme activities were evident in CuONPs-exposed groups (*p* < 0.05). Fish exposure to 50 mg/L CuONPs significantly upregulated the transcription of pro-inflammatory cytokines (tumor necrosis factor-alpha, interleukin-1beta, interleukin 12, and interleukin 8), heat shock protein 70, apoptosis-related gene (caspase 3), and oxidative stress-related (superoxide dismutase, catalase, and glutathione peroxidase) genes in liver and gills of the exposed fish in comparison with those in the control group (*p* < 0.05). Moreover, varying histopathological injuries were noticed in the hepatopancreatic tissues, posterior kidneys, and gills of fish groups correlated to the tested exposure dose of CuONPs. In summary, our results provide new insights and helpful information for better understanding the mechanisms of CuONPs toxicity in Nile tilapia at hematological, molecular levels, and tissue levels.

## 1. Introduction

A wide range of nanotechnology-based industrial products and applications have existed since the beginning of the nanotechnology industry [1,2]. Engineered metal oxide nanomaterials (MONMs) are increasingly manufactured because of their vast array of applications in various industrial products [3]. From MONMs, copper oxide nanoparticles (CuONPs) have gained great interest because of their wide range of beneficial biomedical applications [4], diagnostic imaging [5], catalytic, electric, and optical properties [6,7], and biocidal, antimicrobial, and antifungal activities [8,9]. Besides, they are also present in numerous industrial applications, including sensors [10], antifouling paints [11], and printing inks [12].

The widespread use of MONMs may end up in the aquatic environment causing serious concern and hazardous effects on the exposed aquatic biota, including fish and bivalve mollusks [13,14,15]. In this concern, Malhotra et al. [16] reviewed the nanotoxicological investigations of CuONPs in various fish species, including their bioavailability, bioaccumulation, mechanisms of action, and health effects on the exposed fish. Previous studies showed that the toxicological effects of CuONPs in fish are generally affected by the particle size and the application method [17] and the agglomeration, dissolution, and concentration of nanoparticles in the exposure media [18].

Reports showed that the exposure of the fish to CuONPs caused oxidative stress damage and teratogenicity in zebrafish (*Danio rerio*) embryos [19], haemato-biochemical alterations in Caspian trout (*Salmo trutta caspius*) [20], oxidative stress and higher accumulation of copper in the liver and muscular tissues of African catfish (*Clarias gariepinus*) [21,22]. Moreover, CuONPs induced serious histopathological alterations in organs of the exposed rainbow trout (*Oncorhynchus mykiss*) [23], common carp (*Cyprinus carpio*) [24,25], guppy (*Poecilia reticulata*) [26], and recently, Streaked prochilod (*Prochilodus lineatus*) [27].

At the molecular levels, CuONPs exposure induces modulation of the transcriptomic responses of the immune genes in the intestine of orange-spotted grouper (*Epinephelus coioides*) juveniles [28], proteins associated with oxidative stress in the liver of common carp juveniles [29], heat shock protein 70 (*HSP70*), and pro-inflammatory cytokine genes of tissues zebrafish embryos [30], *HSP70*, *HSP90*, and lysozyme genes in the liver of Puffer fish (*Takifugu fasciatus*) juveniles [31], and recently apoptosis-related genes common carp larvae [32].

Globally, Nile tilapia (*Oreochromis niloticus*) is a good candidate for freshwater culture because of its high market value, consumer preferences, fast growth, ability to grow at different culture systems, and being somewhat tolerant to poor environmental conditions [33,34]. Moreover, Nile tilapia can be regarded as a well-established fish model for toxicological studies because of its simple handling, maintenance under laboratory conditions, and prompt response to pollutants and various toxicants [35,36]. Reports showed that the exposure of Nile tilapia to CuONPs caused oxidative stress damage [37,38], histopathological alterations [39], higher accumulation of copper in the liver [40], haemato-biochemical changes [41], and alterations of liver and kidney functions [42].

No available information was published on the mechanisms of CuONPs toxicity in Nile tilapia at the molecular levels. In this context, the present study describes the sub-lethal effects of waterborne exposure of CuONPs on serum biochemical indices, histopathological alterations of the exposed Nile tilapia and the transcriptomic profile analysis of *HSP70*, pro-inflammatory cytokines, apoptosis- and oxidative stress-related genes in gills and liver. The findings of this study provide new insights and additional information for better elucidation of the mechanisms of CuONPs toxicity in Nile tilapia by assessing the tissue histomorphological criteria, serum biochemistry, and molecular parameters, including gene transcriptions.

## 2. Materials and Methods

### 2.1. Characterization of Nanoparticles

Copper oxide nanoparticles (CuONPs) powder was commercially purchased from Naqaa Nanotechnology Co., Cairo, Egypt, and was synthesized according to the methods described by Khan et al. [43]. The morphology and particle size of CuONPs was determined at 120 KV by transmission electron microscopy (TEM) (JEM-1400, JEOL Ltd., Tokyo, Japan). Surface characterization on the synthesized CuONPs was demonstrated by using scanning electron microscopy (SEM) and energy-dispersive X-ray spectroscopy (EDX) (JSM-5300, JEOL Ltd., Tokyo, Japan). TEM, SEM, and EDX procedures were done at the Electronic Microscope Unit, Faculty of Science, Alexandria University, Egypt. Zeta potentials of CuONPs was demonstrated in deionized water using Zetasizer Nano Series (Model 1801102S, Malvern Instruments, Malvern, UK) at the Central Laboratory, Faculty of Pharmacy, Alexandria University, Alexandria, Egypt.

### 2.2. Fish Acclimation, Maintenance, and Rearing Conditions

Nile tilapia (*Oreochromis niloticus*) juveniles (*n* = 380), with an average initial body weight of 21.50 ± 0.5 g and an average initial length of 12.0 ± 2.5 cm, were purchased from a local fish hatchery, Behera province, Egypt. Fish were transferred to the wet laboratory and stocked for 14 days in six 500 L rearing tanks to be acclimated to the laboratory conditions. Fish were fed ad libitum to apparent satiation three times per day on a well-balanced commercial pellet diet (Aller Aqua Co., October, Egypt) containing 30% protein and all the requirements for optimal fish growth according to the guidelines of NRC [44].

During acclimation, to ensure healthy and safe rearing conditions, 1/3 of the water in each aquarium was daily siphoned and then substituted with new water from the storage tank to minimize the contamination from the uneaten feed and reduce the metabolic wastes. During the experimental study, fish were reared in 100 L glass aquaria supplied with fresh de-chlorinated and well-aerated tap water supplied with compressed air via air stones using air pumps. The light was adjusted at 12 h light: 12 h dark cycle by fluorescent light tubes. The physical and chemical properties of the water were maintained during the present study for temperature (28.0 ± 0.5 °C), pH value (7.60 ± 0.6), dissolved oxygen (7.85 ± 0.46 mg/L), nitrite (0.007 mg/L), total hardness (155.5 mg CaCO_3_/L), un-ionized ammonia (0.014 ± 0.03 mg/L), and total alkalinity (17.83 mg/L) [45].

### 2.3. Ethical Approval

Exposure experiments in the present study were done following the guidelines demonstrated by the Local Experimental Animal Care Committee and approved by the Institutional Ethics Committee of Faculty of Veterinary Medicine, Alexandria University, Egypt (Approval no. 203542).

### 2.4. Preparation of CuONPs Stock Solution

A stock solution of CuONPs was prepared by dispersing the NPs in ultra-pure water (Milli-Q type 1 Ultrapure Water Purification Systems) (Millipore Co., Billerica, MA, USA) with ultrasonication for 1 h in a bath-type sonicator (100 W/L, 40 kHz) to increase the dispersion of the NPs. The procedure of ultrasonication was done 20 min before the daily dosing.

### 2.5. Experimental Setup and CuONPs Exposure

#### 2.5.1. The 96-h Acute Toxicity Test

A total number of 160 Nile tilapia juveniles were grouped into eight groups (each group contains 20 fish) and were exposed to different levels of CuONPs (0.0, 25, 50, 75, 100, 125, 150, and 175 mg/L). Fish were observed for 96 h to calculate the median lethal dose concentrations (LC_50_) of CuONPs according to Finney’s probit analysis [46]. The 96 h LC_50_ value was 100 mg/L (Please see Supplementary Materials Table S1).

#### 2.5.2. Sub-Acute Toxicity Test

Nile tilapia juveniles (*n* = 120) were randomly allotted into four experimental groups in triplicates, and each replicate contains ten fish to ensure the possible reproducibility of the results. Based on the 96h LC_50_ results, waterborne exposure was done using sub-lethal doses (1/2, 1/5, and 1/10 of the 96 h LC_50_ value), which are corresponding to 50, 20, and 10 mg/L, respectively, were used for the sub-acute toxicity study.

Groups I, II, and III were exposed to 10 mg/L, 20 mg/L, and 50 mg/L of the CuONPs solution. Group IV was maintained in de-chlorinated tap water without CuONPs and served as a control group. The experiment was continued for 25 days. During the exposure period, the tested solution dose per each experimental group was daily calculated and replenished to maintain the relative constant concentrations and dispersity of CuONPs.

Moreover, a semi-static water flow regime was followed (whereas 50% of the water was daily exchanged from all aquaria and renewed with well-aerated water from the storage tank before the re-dosing of CuONPs), and the exposure doses of CuONPs were calculated after each water exchange. To ensure the dispersion of the used CuONPs dose, ultrasonication was done 20 min daily before re-dosing (Please see Section 2.4. Preparation of CuONPs stock solution).

Importantly, the disposal of wastewater after water exchange was completely done under strict hygienic measures to avoid environmental pollution. The feeding regime was noteworthily done after each water change before re-dosing of CuONPs to reduce the risk of ingestion of CuONPs during feeding.

### 2.6. Sample Collection

Fish were starved 24 h before sampling. Then, fish were anesthetized by 100 µg/mL buffered tricaine methane sulphonate (MS-222) (Finquel, Argent Chemical Laboratories, Redmond, WA, USA) for collection of blood samples.

#### 2.6.1. Serum Samples

Nine fish per each experimental group (*n* = 9) (three fish from each replicate) were sampled. Blood was sampled from the caudal veins using a 1 mL syringe. Blood samples were left at room temperature to clot for collection of the serum. Sera samples were separated by centrifugation (3000× *g* for 15 min) into a centrifuge tube and then stored at −20 °C until used in serum measurements.

#### 2.6.2. Tissue Samples

For collection of tissue samples, fish were euthanized with overdosage of MS-222. Tissue specimens (*n* = 9 fish per group) were collected from the liver, gills, and kidneys of the control and experimentally exposed fish for histopathological studies. Additionally, gills and liver specimens (*n* = 9 fish per group) were collected, immediately frozen in liquid nitrogen, and then stored at −80 °C until used for gene expression assays.

### 2.7. Serum Biochemical Indices

Blood urea nitrogen and creatinine levels were estimated using fish-specific kits (Bio diagnostic Co., Cairo, Egypt) according to Coulombe and Favreau [47] and Larsen [48], respectively. Serum alanine transaminase (ALT), aspartate transaminase (AST), and alkaline phosphatase (ALP) activities were determined calorimetrically according to Reitman and Frankel [49] and Tietz et al. [50] respectively, by using fish-specific kits (Bio diagnostic Co., Cairo, Egypt) according to the guidelines from the manufacturer.

### 2.8. Gene Transcription

Total RNA was extracted from the gills and liver tissues (100 mg per each) and was used for real-time PCR (RT-PCR). Total RNA was prepared using Trizol reagent (iNtRON Biotechnology Inc., Seongnam, Gyeonggi-do, Korea) following the manufacturer’s instructions. The quantity of the extracted RNA was confirmed by Nanodrop (Uv–Vis spectrophotometer Q5000/Quawell, San Jose, CA, USA). Afterward, the complementary DNA (cDNA) was synthesized using the SensiFAST™ cDNA synthesis kit (Bioline/Meridian Bioscience, London, UK) following the manufacturer’s instructions. The cDNA samples were then stored at −20 °C until use.

Table 1 shows the specific primer sequences and GenBank accession numbers of target genes used in the current investigation, which include heat shock protein 70 (HSP70), apoptosis-related gene (caspase 3), oxidative stress genes such as superoxide dismutase (SOD), glutathione peroxidase (GPX), and catalase (CAT), and cytokine genes such as tumor necrosis factor-alpha (TNF-α), interleukin 1 beta (IL-1β), interleukin 8 (IL-8), and interleukin 12 (IL-12), and interleukin 10 (IL-10). Moreover, beta-actin (β-actin) was used as a housekeeping gene (as a reference) to quantify the mRNA expression folds in the tested fish tissues.

The SYBR green method was used to quantify the mRNA expression folds using RT-PCR (SensiFast SYBR Lo-Rox kit, Bioline/Meridian Bioscience, London, UK). The thermocycling conditions for the reaction were 10 min (at 95 °C), followed by 40 cycles of 15 s (at 95 °C), 30 min (at 60 °C), and finally 5 min (at 85 °C) for 1 min. The runs were conducted, and the mRNA expression folds were standardized to the β-actin mRNA transcripts using the 2^−ΔΔCT^ method, according to Schmittgen and Livak [51]. 

### 2.9. Histopathological Studies

The collected liver, gills, and kidney specimens were washed with sterile saline solution and then directly fixed in 10% buffer formalin solution for 48 h. The fixed specimens were then processed using the paraffin embedding technique [52]. Specimens were dehydrated in ascending concentrations of ethanol, cleared in xylene, blocked in paraffin wax, sectioned (multiple 5–8 μm thickness sections) using an ultra-microtome (Leica Microsystems, Wetzlar, Germany), and finally stained with hematoxylin and eosin (H & E) stain [53]. Representative photomicrographs were then captured from the prepared tissue sections using a digital camera (Leica EC3, Leica, Wetzlar, Germany) connected to a microscope (Leica DM500, Wetzlar, Germany) to demonstrate the histopathological alterations that occurred in the examined fish tissues after exposure to CuONPs compared to the control group.

### 2.10. Statistical Analysis

Data are presented as means ±the standard error of means. All data were examined for the normality and homogeneity of variances using the Kolmogorove–Smirnov test and Levene’s test. One-way analysis of variance (ANOVA) was performed using the SPSS program (version 22.0; SPSS Inc., Chicago, IL, USA) and GraphPad Prism Software (version 5) (GraphPad Software, San Diego, CA, USA. Duncan’s multiple range test was used to determine the individual comparisons between the CuONPs-exposed groups and the control group. *p* < 0.05 was considered to be statistically significant.

## 3. Results

### 3.1. Characterization of CuONPs

The characteristics of the CuONPs sample used in the present study (please see Supplementary Materials) showed that the TEM images declared the morphological information of CuONPs had an irregular nanorod shaped particles with a relatively uniform size distribution (Figure S1) with an average size distribution was 68.92 ± 3.49 nm. The surface charge of CuONPs in water was measured as a zeta potential of −15.5 mV (Figure S2). Moreover, the spectroscopic composition analysis by EDX demonstrated the presence of copper and oxygen elements in the constituents of the CuONPs sample used in the present study (Figure S3).

### 3.2. Serum Biochemical Indices

Table 2 describes the alterations serum biochemical measurements in the control and CuONPs groups after 25 days of the exposure period. Compared to the control group, there was a significant dose-dependent elevation in blood urea and creatinine levels as well as serum ALT, AST, and ALP enzyme activities in CuONPs-exposed fish groups compared to the control group (*p* < 0.05). Interestingly, the highest blood urea and creatinine levels and serum ALT, AST, and ALP activities were demonstrated in the fish group exposed to 50 mg CuONPs/L.

### 3.3. Gene Transcription

#### 3.3.1. Gill Tissues

**Figure 1:**
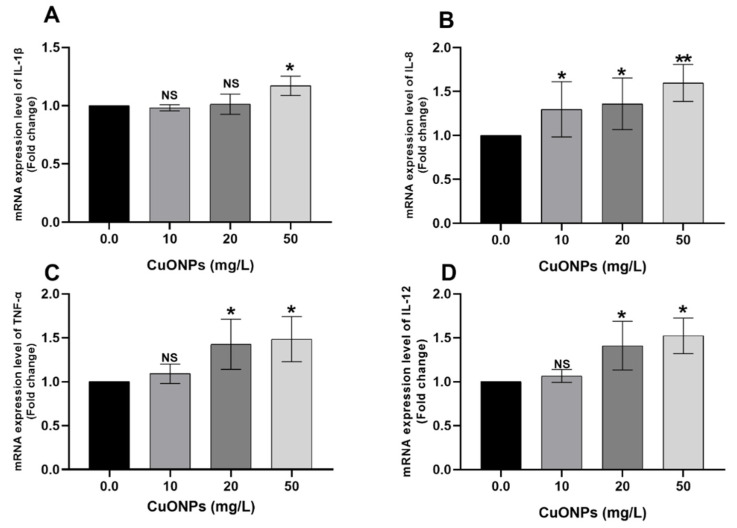
mRNA transcription profile of the pro-inflammatory cytokine genes including *IL-1β* (**A**), *IL-8* (**B**), *TNF-α* (**C**), and *IL-12* (**D**) in the gills of Nile tilapia juveniles after exposure to sub-lethal concentrations of CuONPs (0.0 mg/L, 10 mg/L, 20 mg/L and 50 mg/L) for 25 days. The values are expressed as mean ± SEM (*n* = 9). Asterisk (*) (*p* < 0.05) and (**) (*p* < 0.01) indicates significant differences between the exposure groups compared with the control group. NS indicates non-significant differences.

Figure 1 showed the mRNA transcription profile of the pro-inflammatory cytokine genes in the gills of Nile tilapia juveniles after exposure to sub-lethal concentrations of CuONPs for 25 days compared to the control group. Pairwise comparisons with the control group showed that there was significant upregulation of the *IL-1β* gene (Figure 1A, *p* < 0.05) in the fish group exposed to 50 mg/L CuONPs. Moreover, significant upregulations of *TNF-α* (Figure 1C) and *IL-12* (Figure 1D) genes (*p* < 0.05) were observed in fish groups exposed to 20 and 50 CuONPs mg/L groups. Interestingly, all tested concentrations of CuONPs significantly increased the mRNA expression folds of the *IL-8* gene (Figure 1B; *p* < 0.05) compared to the control group.

**Figure 2:**
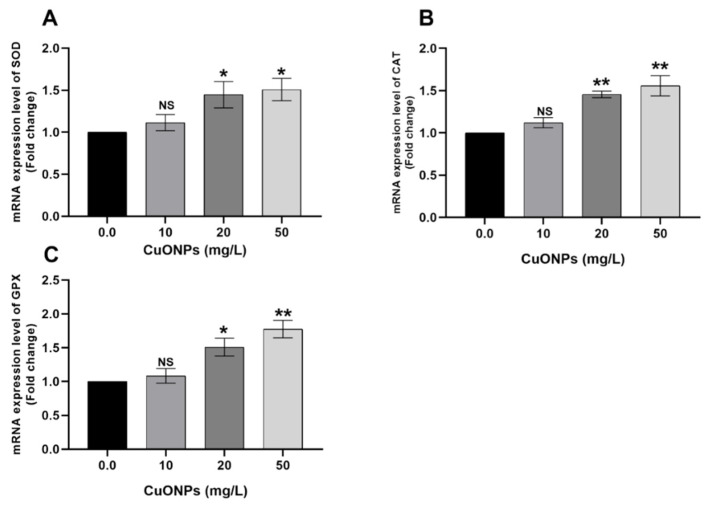
mRNA transcription profile of oxidative stress-related genes including *SOD* (**A**), *CAT* (**B**), and *GPX* (**C**) in the gills of Nile tilapia juveniles after exposure to sub-lethal concentrations of CuONPs (0.0, 10, 20, and 50 mg/L) for 25 days. The values are expressed as mean ± SEM (*n* = 9). Asterisk (*) (*p* < 0.05) and (**) (*p* < 0.01) indicate significant differences between the exposure groups compared with the control group. NS indicates non-significant differences.

Figure 2 showed the mRNA transcription profile of oxidative stress-related genes in the gills of Nile tilapia juveniles after exposure to sub-lethal concentrations of CuONPs for 25 days compared to the control group. There were significant upregulations of the *SOD* (Figure 2A), *CAT* (Figure 2B), and *GPX* (Figure 2C) genes (*p* < 0.05) in fish groups exposed to 20 and 50 mg/L CuONPs compared to the control group.

**Figure 3:**
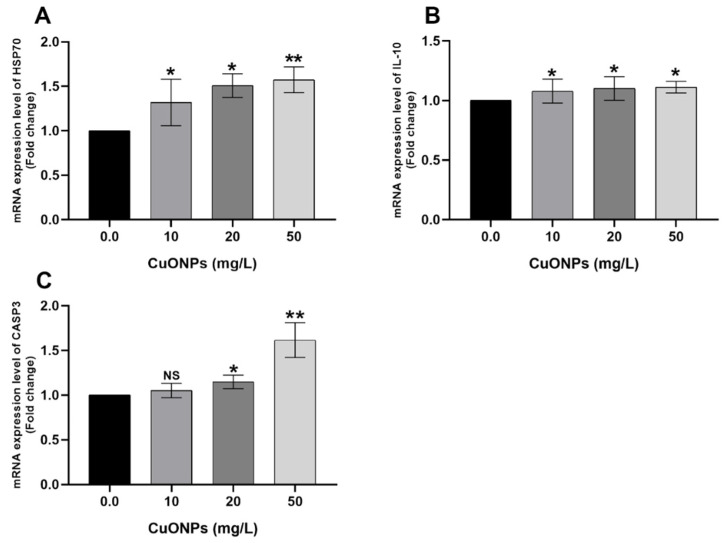
mRNA transcription profile of *HSP70* (**A**), *IL-10* (**B**), and *CASP3* (**C**) genes in the gills of Nile tilapia juveniles after exposure to sub-lethal concentrations of CuONPs (0 mg/L, 10 mg/L, 20 mg/L and 50 mg/L) for 25 days. The values are expressed as mean ± SEM (*n* = 9). Asterisk (*) (*p* < 0.05) and (**) (*p* < 0.01) indicate significant differences between the exposure groups compared with the control group. NS indicates non-significant differences.

On the other hand, all tested levels of CuONPs significantly increased the mRNA expression folds of *HSP70* (Figure 3A) and *IL-10* (Figure 3B) genes in the gills of the exposed fish (*p* < 0.05) compared to the control group. Moreover, the mRNA expression levels of *CASP3* gene (Figure 3C) were significantly increased in 20 mg/L CuONPs group (*p* < 0.05) and in 50 mg/L CuONPs group (*p* < 0.01) compared to the control group.

#### 3.3.2. Hepatic Tissues

**Figure 4:**
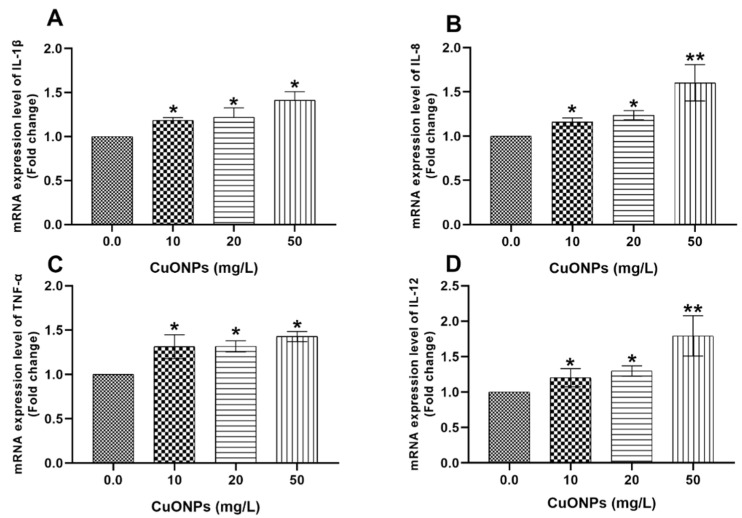
mRNA transcription profile of pro-inflammatory cytokine genes including *IL-1β* (**A**), *IL-8* (**B**), *TNF-α* (**C**), and *IL-12* (**D**) genes in the hepatic tissues of Nile tilapia juveniles after exposure to sub-lethal concentrations of CuONPs (0.0, 10, 20 mg/L and 50 mg/L) for 25 days. The values are expressed as mean ± SEM (*n* = 9). Asterisk (*) (*p* < 0.05) and (**) (*p* < 0.01) indicate significant differences between the exposure groups compared with the control group.

**Figure 5:**
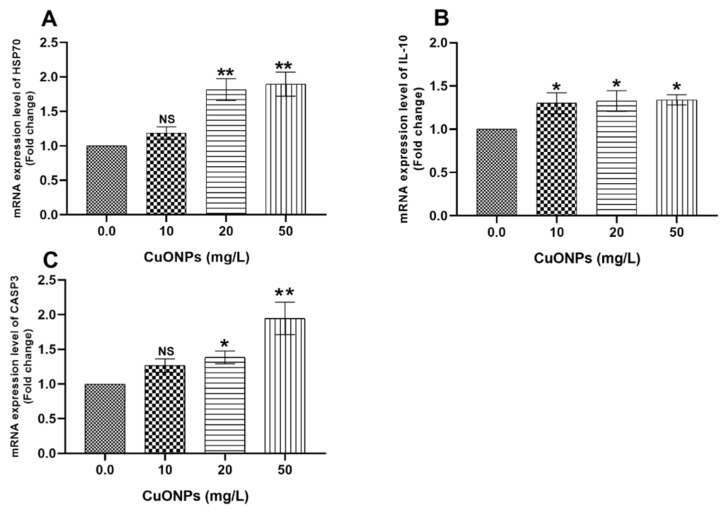
mRNA transcription profile of HSP70 (**A**), IL-10 (**B**), and CASP3 (**C**) genes in the hepatic tissues of Nile tilapia juveniles after exposure to sub-lethal concentrations of CuONPs (0.0 mg/L, 10 mg/L, 20 mg/L and 50 mg/L) for 25 days. The values are expressed as mean ± SEM (*n* = 9). Asterisk (*) (*p* < 0.05), and (**) (*p* < 0.01) indicate significant differences between the exposure groups compared with the control group. NS indicates non-significant differences.

All tested sub-lethal doses of CuONPs significantly increased the mRNA expression folds of *IL-1β* (Figure 4A), *IL-8* (Figure 4B), *TNF-α* (Figure 4C), *IL-12* (Figure 4D), and *IL-10* (Figure 5B) genes (*p* < 0.05) in the hepatic tissues of Nile tilapia juveniles compared to the control group. 

**Figure 6:**
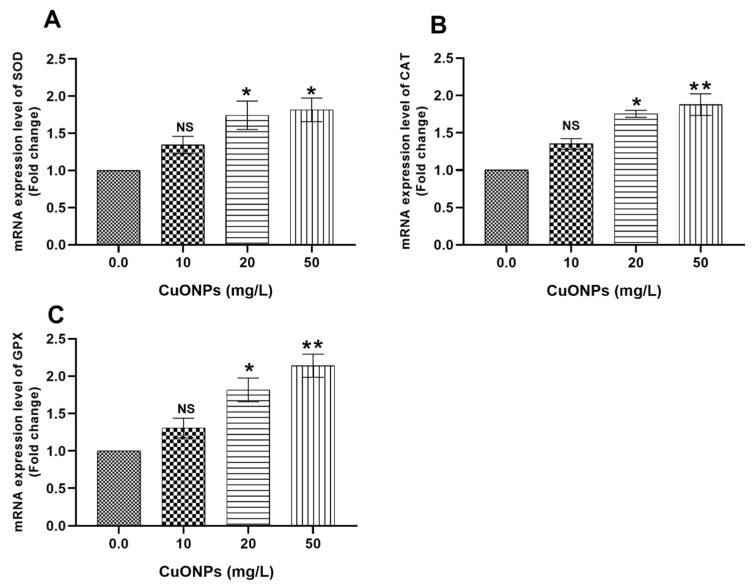
mRNA transcription profile of oxidative stress-related genes (**A**) SOD, (**B**) CAT, and (**C**) GPX in the hepatic tissues of Nile tilapia juveniles after exposure to sub-lethal concentrations of CuONPs (0.0 mg/L, 10 mg/L, 20 mg/L and 50 mg/L) for 25 days. The values are expressed as mean ± SEM (*n* = 9). Asterisk (*) (*p* < 0.05), and (**) (*p* < 0.01) indicate significant differences between the exposure groups compared with the control group. NS indicates non-significant differences.

Moreover, significant upregulations of *SOD* (Figure 6A), *CAT* (Figure 6B), *GPX* (Figure 6C), *HSP70* (Figure 5A), and *CASP3* (Figure 5C) genes (*p* < 0.05) were observed in the hepatic tissues of fish reared at water polluted with 20 mg/L and 50 mg/L CuONPs compared to the control group.

### 3.4. Histopathological Alterations

#### 3.4.1. Gills

**Figure 7:**
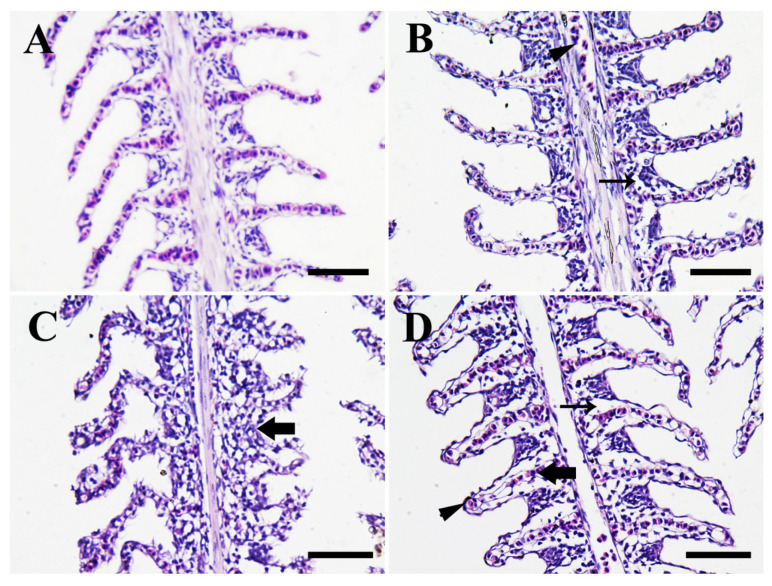
Representative photomicrographs in the gills of Nile tilapia juveniles (H & E stain, scale bar = 50 μm) of the control group (**A**), and CuONPs-exposed fish at 10 mg/L (**B**), 20 mg/L (**C**), and 50 mg/L (**D**) values, respectively for 25 days. (**A**) shows normal gill architecture, primary and secondary gill lamellae. Meanwhile, (**B**) shows slight dilation of primary and secondary lamellae due to edema (arrow) and slight congestion (arrowhead). (**C**) shows epithelial necrosis, desquamation, fusion, and epithelial layer rupture (arrow), and (**D**) shows severe edema (thin arrow), sever congestion of secondary lamellae (thick arrow), and telangiectasis of the secondary lamellae (arrowhead).

Figure 7 shows the photomicrographs in the gills of Nile tilapia in the control group compared to CuONPs-exposed fish groups for 25 days. Fish in the control group (Figure 7A) showed normal histological structure of the gill architecture, primary and secondary gill lamellae. On the other hand, gills of CuONPs-exposed fish showed dose-dependent histopathological alterations ranged from slight dilation of primary and secondary gill lamellae due to edema and slight congestion (10 mg/L CuONPs; Figure 7B), epithelial necrosis, desquamation, fusion, and epithelial layer rupture (20 mg/L CuONPs; Figure 7C), and severe edema, congestion, and telangiectasis of the secondary gill lamellae (50 mg/L CuONPs; Figure 7D).

#### 3.4.2. Hepatopancreatic Tissues

**Figure 8:**
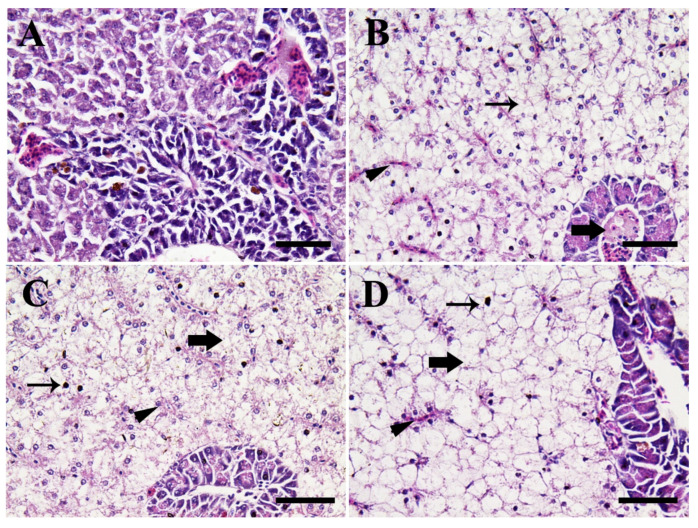
Representative photomicrographs in the hepatic tissues of Nile tilapia juveniles (H & E stain, scale bar = 50 μm) of the control group (**A**), and CuONPs-exposed fish at 10 mg/L (**B**), 20 mg/L (**C**), and 50 mg/L (**D**) values, respectively for 25 days. (**A**) shows normal hepatopancreatic architecture with a normal hepatic cord and acini of the exocrine pancreas. Meanwhile, (**B**) shows slight vascular congestion (arrowhead), diffuse fatty vacuolization in hepatocytes (thin arrow), and infiltration of mononuclear inflammatory cells was evident particularly within pancreatic acinar cells (thick arrow). (**C**) shows moderate congestion (arrowhead), diffuse fatty vacuolized hepatocytes (thick arrow), and moderate necrosis (thin arrow), and (**D**) shows severe necrosis (thin arrow), fatty degeneration of hepatocytes (thick arrow), and severe congestion of blood sinusoid (arrowhead).

Figure 8 shows the photomicrographs in the hepatopancreatic tissues of Nile tilapia in the control group compared to CuONPs-exposed fish groups for 25 days. Fish in the control group showed normal hepatopancreatic architecture with normal hepatic cord and acini of the exocrine pancreas (Figure 8A).

The histopathological alterations of the hepatopancreatic tissues in fish groups exposed to sub-lethal levels of CuONPs showed dose-dependent alterations ranged from slight vascular congestion, diffuse fatty vacuolization in hepatocytes which contain centrally eccentrically necrotic nucleus, and infiltration of mononuclear inflammatory cells within the pancreatic acinar cells (10 mg/L CuONPs; Figure 8B), moderate congestion, diffuse fatty vacuolized hepatocytes and moderate necrosis (20 mg/L CuONPs; Figure 8C), and severe necrosis, fatty degeneration of hepatocytes and severe congestion of blood sinusoids (50 mg/L CuONPs; Figure 8D).

#### 3.4.3. Kidneys

**Figure 9:**
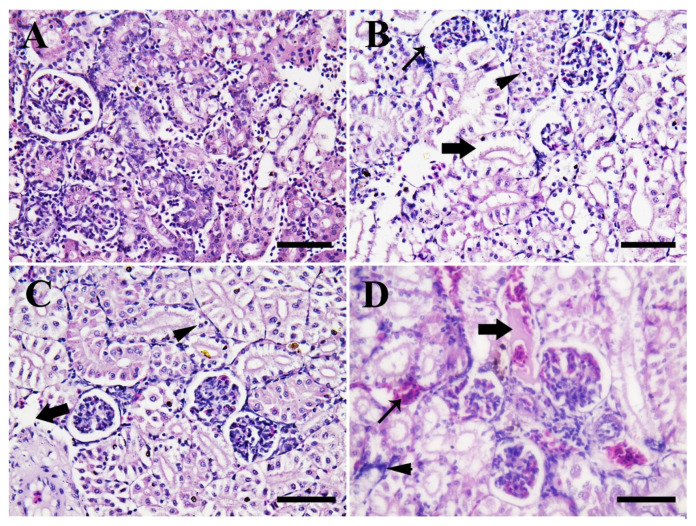
Representative photomicrographs in the posterior kidneys of Nile tilapia juveniles (H & E stain, scale bar = 50 μm) of the control group (**A**), and CuONPs-exposed fish at 10 mg/L (**B**), 20 mg/L (**C**), and 50 mg/L (**D**) values, respectively for 25 days. (**A**) shows normal renal architecture that consisted of renal tubules, renal lining epithelium, glomerulus, and bowman’s spaces. Meanwhile, (**B**) shows widened bowman spaces (thin arrow), widened renal tubules with slight necrosis of several renal tubules and edema (thick arrow), and pyknotic nuclei (arrowhead). (**C**) shows moderate necrosis of many renal tubules and edema (thick arrow), moderate distribution of pyknotic nuclei (arrowhead), and (**D**) shows intraluminal eosinophilic proteinaceous materials (thick arrow), inter-tubular congestion, multiple focal areas of inter-tubular hemorrhage (thin arrow), and inflammatory cells were widely distributed (arrowhead).

Figure 9 shows the photomicrographs in the posterior kidney of Nile tilapia in the control group compared to CuONPs-exposed fish groups for 25 days. Fish in the control group showed normal renal architecture (Bowman’s spaces, tubules, epithelial lining, and glomeruli) (Figure 9A). The histopathological alterations of the posterior kidneys in fish exposed to 10 mg/L CuONPs showed widened Bowman’s spaces and renal tubules alongside slight necrotic changes were evident in multiple renal tubules, edema, and pyknotic nuclei were evident (Figure 9B).

Posterior kidneys of fish exposed to 20 mg/L CuONPs showed moderate necrosis of many renal tubules, edema, and pyknotic nuclei (Figure 9C). Moreover, posterior kidneys of fish exposed to 50 mg/L CuONPs showed inter-tubular congestion, intra-luminal eosinophilic proteinaceous materials, multiple focal inter-tubular hemorrhage, and inflammatory cells were widely distributed (Figure 9D).

## 4. Discussion

### 4.1. Serum Biochemical Indices

The present investigation showed a dose-dependent increase in blood urea and creatinine, and serum ALT, AST, and ALP enzyme activities in CuONPs-exposed groups compared to the control group (*p* < 0.05) after a long-term exposure period (25 days). Consistent with our findings, Abdel-Khalek et al. [37] described a significant elevation of uric acid and creatinine values, ALP, ALT, and AST activities in Nile tilapia exposed to 15 or 7.5 mg/L CuONPs after long term water exposure (30 days). In a similar sense, blood urea, creatinine, ALP, ALT, and AST values were significantly increased in Nile tilapia exposed for 14 days to different sub-lethal levels of CuONPs [42]. Moreover, Kaviani et al. [20] showed a similar increase in ALP and AST activities in Caspian trout exposed to sub-lethal levels of CuONPs for 28 days. Similarly, Abdel-Daim et al. [14] reported a similar increase in blood urea, creatinine, ALP, AST, and ALT values in Nile tilapia exposed to sub-lethal doses of zinc oxide NPs for 30 days. The increase of blood urea and creatinine levels indicates kidney dysfunction, which might be closely associated with the impairment of renal tubular functions and insufficiency of the glomerular infiltration [14]. Moreover, the release of ALT, ALP, and AST enzymes into the bloodstream and subsequent increase of their serum levels are considered bioindicators of hepatic damage and hepatitis following exposure to pollutants and aquatic toxicants [54,55].

### 4.2. Gene Transcriptions

#### 4.2.1. Cytokines

In the present study, there were significant upregulations of *IL-1β*, *IL-8*, *TNF-α*, and *IL-12* genes was noticed in gills (Figure 1) and liver (Figure 4) of Nile tilapia exposed to 50 mg/L CuONPs for 25 days compared to the control group. Aksakal and Ciltas [30] reported a significant increase of the mRNA expression values of the *IL-1β* gene in tissues of zebrafish embryos exposed to CuONPs. Moreover, a significant increase of the mRNA expression levels of the *IL-1β* and *TNF-α* genes was recorded in the intestine of *E. coioides* juveniles exposed to CuONPs [28]. In a similar sense, increased expression of *IL-6*, *IL-1β*, and *TNF-α* genes in the intestines of gilthead seabream (*Sparus aurata*) exposed to 50 μg/L gold NPs [56].

Notably, it was known that tumor necrosis factors such as *TNF-α* are involved in inflammation, apoptosis, and cell proliferation [57]. Moreover, the pro-inflammatory cytokines such as IL-1β, IL-8, TNF-α, and IL-12 are molecules that play crucial roles in hematopoiesis and inflammatory responses of fish [58,59,60,61]. Thus, our results suggested an increased pro-inflammatory responses that would be directly connected with the responses observed in the liver and gills as a potential immune regulatory mechanism.

IL-10 is anti-inflammatory cytokine [61], and its increased expression in tissues of Nile tilapia exposed to CuONPs hypothesized that fish were exposed to inflammatory responses during exposure. Moreover, it can also be linked to preventive mechanisms exerted by the fish body in response to the exposure to CuONPs toxicity.

#### 4.2.2. Oxidative Stress-Related Genes

Oxidative stress occurred because of the overproduction of free radicals such as reactive oxygen species (ROS), which subsequently trigger the antioxidant defensive mechanisms of the body to overcome the toxic effects of these radicals such as induction of glutathione reductase (GR), glutathione-S-transferase (GST), CAT, SOD, and GPX enzymes and other compounds that involved in the antioxidant defense (e.g., glutathione) [14]. In the current study, significant upregulations of *SOD*, *CAT*, and *GPX* genes were noticed in gills (Figure 2) and liver (Figure 6) of Nile tilapia exposed to 20 mg/L and 50 mg/L CuONPs for 25 days compared to the control group. These findings suggest that CuONPs exposure induced oxidative stress in the exposed fish; meanwhile, the expression of the antioxidant genes in gills and liver suggests the normal protective responses of fish to counteract and mitigate the effects of oxidative damage exerted by CuONPs in their tissues.

In a similar sense, significant upregulations of *SOD1*, *CAT*, and *GPX1a* genes were reported in zebrafish embryos exposed to zinc oxide NPs (ZnONPs) [62]. Moreover, Saddick et al. [63] demonstrated significant upregulation of *CAT*, *SOD*, *GR*, *GST*, and *GPX* genes in the brain tissues of Nile tilapia and *Tilapia zillii* exposed to 500 µg/L ZnONPs. Furthermore, the mRNA expression values of *SOD* and *CAT* genes were significantly upregulated in zebrafish exposed to titanium dioxide NPs [64].

#### 4.2.3. HSP70

In the current study, significant upregulation of *HSP70* was noticed in gills (Figure 3A) and liver (Figure 5A) of Nile tilapia exposed to 20 mg/L and 50 mg/L CuONPs for 25 days compared to the control group. Parallel to our findings, Wang et al. [28] reported significant upregulation of *HSP70* and *HSP90* in the intestines of *E. coioides* juveniles exposed to CuONPs. Aksakal and Ciltas [30] also demonstrated significant upregulation of *HSP70* in zebrafish embryos exposed to CuONPs. Moreover, a significant upregulation in the mRNA expression values of *HSP70* and *HSP90* genes was noticed in the liver of *T. fasciatus* after exposure to CuONPs [31]. Noteworthily, the expression of heat shock proteins (HSPs) such as HSP70 and HSP90 is closely related to fish exposure to stressors [65,66,67]. Besides, HSP70 and HSP90 play pivotal roles in regulating apoptosis through inhibition of the apoptotic cell signal cascade [68]. Therefore, the findings of our study suggest that the expression of *HSP70* in fish tissues elucidates that fish were stressed after exposure to sub-lethal concentrations of CuONPs and might be important for the prevention of the apoptotic changes and cellular signaling in Nile tilapia exposed to the stress effects of CuONPs.

#### 4.2.4. CASP3

Caspases are useful indicators and mediators for detection of stress-induced apoptosis (programmed cell death) [69]. Moreover, *CASP3* is also an indicator of DNA damage and several morphological alterations associated with apoptosis [70]. In the present study, significant increase of the mRNA expression values of *CASP3* gene was noticed in gills (Figure 3C) and liver (Figure 5C) of Nile tilapia exposed to 20 mg/L and 50 mg/L CuONPs for 25 days compared to the control group. Therefore, our findings suggest that CuONPs-induced apoptosis in tissues of the exposed Nile tilapia. The activities of caspase-3 and caspase-9 were considerably increased in the intestines of *E. coioides* juveniles exposed to CuONPs [28]. Moreover, a noticeable increase in the mRNA expression levels of *CASP3* and *CASP9* genes in the liver of *T. fasciatus* after exposure to CuONPs [31]. The mRNA expression levels of *CASP3*, *CASP8*, and *CASP9* genes were appreciably increased in gills of the Japanese rice fish (*Oryzias latipes*) exposed to Multiwall carbon nanotubes for 14 days [71].

### 4.3. Histopathological Alterations

#### 4.3.1. Gills

In the present study, the histopathological alterations in the gills of fish exposed to sub-lethal levels of CuONPs for 25 days showed varying degrees of congestion, epithelial necrosis, desquamation, fusion, rupture, hyperplasia, and edema of the primary and secondary gill lamellae corresponding to the exposure dose of CuONPs (Figure 7). Similar findings were reported in Nile tilapia exposed to sub-lethal doses of CuONPs [39]. Moreover, our findings were also consistent with those reported in the gill tissues of guppies exposed to CuONPs for 10 days [26]. Al-Bairuty et al. [23] illustrated that exposure to CuONPs resulted in edema, hyperplasia, lamellar fusions, clubbed tips, aneurisms, and necrotic changes in the secondary gill lamellae of rainbow trout. Moreover, edema of the gill epithelium, hyperplasia at the base of the secondary gill lamellae, clubbed tips, lamellar fusion, occasional aneurism, and swelling in the secondary lamellae were evident in common carp exposed to CuONPs [29]. Hypertrophy, lamellar fusions, hyperplasia, epithelial lifting, and erythrocyte infiltrations also found in gills of common carp exposed to CuONPs [25]. Besides, shortening of the primary gill lamellae and fusions of the secondary gill lamellae was evident in the Siberian sturgeon (*Acipenser baerii*) after CuONPs exposure for 21 days [72]. Recently, hyperplastic changes of the gill filaments were demonstrated in *P. lineatus* exposed to waterborne exposure to CuONPs [27].

Fish gills are continuously in direct contact with the water pollutants because of their anatomical structure. Thus, the defensive mechanisms of the gills against any water pollutants started by excessive secretion of mucous from the goblet cells [73]. Consequently, edema, lifting of the lamellar epithelium, and epithelial hyperplasia occurred for a trial to inhibit the toxin entry to the exposed fish [74]. Hyperplasia leads to lamellar fusion, which will counteract the gaseous exchange mechanisms, negatively affect the oxygen uptake by the gill tissues, and subsequently increased the partial pressure of carbon dioxide leading to metabolic and respiratory acidosis [75]. The evidence of gill edema may be closely linked to the toxic effects of NPs in the inhibition of ionic transport across the gill epithelium by the branchial Na^+^/K^+^ ATPase, which will, in turn, leads to disruption of the osmotic regulation and then osmotic imbalance occurs [23,76]. Collectively, the exposure to CuONPs might induce osmoregulatory failure, negatively affect the countercurrent gaseous exchange mechanisms, and decrease the uptake of dissolved oxygen from the water, which will subsequently lead to asphyxiation, respiratory failure, and death [77,78].

#### 4.3.2. Hepatopancreatic Tissues

The liver is the main organ of detoxification of xenobiotics, and histopathological alterations in the hepatopancreatic tissues are often linked with the exposure of fish to aquatic pollutants [55,79]. In the present investigation, the histopathological alterations of the hepatopancreatic tissues in Nile tilapia groups exposed to sub-lethal levels of CuONPs for 25 days showed necrosis, fatty degeneration of hepatocytes, and severe congestion of blood sinusoids corresponding to the exposure dose (Figure 8). Previous reports illuminated that fish exposed to CuONPs demonstrated hepatic damage [80]. Al-Bairuty et al. [23] reported necrosis, small foci of hepatitis-like injury, pyknotic nuclei, and an increased number of melanomacrophage deposits in CuONPs-exposed common carp. Moreover, Gupta et al. [29] reported a pronounced increased number of pyknotic nuclei, vacuolations, and necrotic changes in hepatopancreatic tissues of common carp exposed to CuONPs. Ostaszewska et al. [72] also reported vacuolation of hepatocytes of Siberian sturgeon exposed to CuONPs. Similarly, degenerative changes and vacuolization of the hepatocytes, pyknotic nuclei, damaged central vein, nuclear hypertrophy, and dilated hepatic sinusoids were noticed in the hepatic tissues of common carp exposed to CuONPs [81]. Recently, hepatic damage was also defined in *P. lineatus* exposed to sub-lethal doses of CuONPs [27]. Noteworthily, the exposure of fish to toxicants and aquatic contaminants may induce disorganization and vacuolization of the hepatocytes, change the shape and size of the nuclei, and focal necrosis [82,83]. Moreover, Al-Bairuty et al. [23] suggest that vacuolation of the hepatocytes in fish exposed to CuONPs toxicity might be linked to bioaccumulation of triglycerides in the hepatocytes.

#### 4.3.3. Posterior Kidneys

In the present study, the posterior kidneys of Nile tilapia exposed to sub-lethal levels of CuONPs showed widened Bowman’s spaces, necrosis of renal tubules, edema, pyknotic nuclei, multiple focal areas of inter-tubular hemorrhage, and infiltration with inflammatory cells in corresponding to the exposure dose (Figure 9). Damage of the epithelial cells of the renal tubules, changes in the Bowman’s spaces, and increase of the melanomacrophage deposits foci were reported in kidneys of rainbow trout exposed to CuONPs [23]. Moreover, Gupta et al. [29] reported tubular necrosis, damage of the epithelial cells of the renal tubules, and increased Bowman’s spaces in common carp exposed to sub-lethal doses of CuONPs. The findings of our study suggest a disturbance of the osmoregulatory mechanisms of fish kidneys. In general, degenerative, and necrotic changes in the renal tubular epithelium may be associated with heavy metals and pesticide toxicity in fish [53,79]. Moreover, the increased Bowman’s spaces may be attributed to impaired glomerular filtration resulting from obstructed tubule lumen because of damaged epithelial cells of the renal tubules [23].

## 5. Conclusions

In summary, data from our study showed that the Nile tilapia exposure to sub-lethal concentrations of CuONPs (10, 20, and 50 mg/L) induced impairment of the liver and kidney functions, injurious histopathological changes, and modulation of the transcriptomic profile in liver and gills during long-term exposure. Collectively, the increase of blood metabolites, the irreversible degenerative alterations in the hepatopancreatic tissues, kidneys, and gills, and upregulation of the mRNA expression values of pro-inflammatory cytokines, *HSP70*, *CASP3*, and oxidative stress-related genes are strong evidence of the occurrence of inflammatory reactions, and oxidative stress damage because of the toxic actions of CuONPs. This study provided detailed outlines and a set of molecular biomarkers for evaluating the toxic effects of CuONPs in the exposed Nile tilapia, which may positively contribute to the knowledge about CuONPs toxicity in aquatic organisms.

## Figures and Tables

**Table 1:** Primers sequences for the target genes used for SYBR green real-time PCR (RT-PCR).

Target mRNA	Primer Sequences (5′–3′) (F: Forward and R: Reverse)	NCBI GenBank Accession No.
*CASP3*	F-GGCTCTTCGTCTGCTTCTGT	GQ421464.1
	R-GGGAAATCGAGGCGGTATCT	
*HSP70*	F-CATCGCCTACGGTCTGGACAA	FJ207463.1
	R-GCCGTCTTCAATGGTCAGGAT	
*SOD*	F-CCCTACGTCAGTGCAGAGAT	JF801727.1
	R-GTCACGTCTCCCTTTGCAAG	
*GPX*	F-CGCCGAAGGTCTCGTTATTT	NM_001279711.1
	R-TCCCTGGACGGACATACTT	
*CAT*	F-CCCAGCTCTTCATCCAGAAAC	JF801726.1
	R-GCCTCCGCATTGTACTTCTT	
*IL-10*	F-CTGCTAGATCAGTCCGTCGAA	XM_003441366.2
	R-GCAGAACCGTGTCCAGGTAA	
*TNF-α*	F-GAAGCAGCTCCACTCTGATGA	JF957373.1
	R-ACAGCGTGTCTCCTTCGTTCA	
*IL-1β*	F-AAGGATGACGACAAGCCAACC	XM_003460625.2
	R-GCGGACAGACATGAGAGTGC	
*IL-8*	F-TCATTGTCAGCTCCATCGTG	NM_001279704.1
	R-CCTGTCCTTTTCAGTGTGGC	
*IL-12*	F-GGGTGCGAGTCAGCTATGAG	XM_003437924.4
	R-GGTTGTGGATTGGTTGCGTC	
*β-actin*	F-CCACACAGTGCCCATCTACGA	EU887951.1
	R-CACGCTCTGTCAGGATCTTCA	

HSP70: Heat shock protein 70, CASP3: Caspase 3, IL-10: Interleukin 10, SOD: Superoxide dismutase, GPX: Glutathione peroxidase, CAT: Catalase, TNF-α: Tumor necrosis factor alpha, IL-1β: Interleukin 1 beta, IL-8: interleukin 8, IL-12: Interleukin 12, β-actin: Beta actin.

**Table 2:** Changes in serum biochemical indices in Nile tilapia juveniles following exposure to sub-lethal levels of CuONPs for a long exposure period (25 days).

Parameters	Copper Oxide Nanoparticles (CuONPs) (mg/L)			
	0.0	10	20	50
Urea (mg/dL)	7.85 ± 0.81 c	9.65 ± 0.39 bc	11.01 ± 0.84 b	14.91 ± 0.88 a
Creatinine (mg%)	0.64 ± 0.06 c	0.99 ± 0.17 b	1.11 ± 0.09 ab	1.87 ± 0.25 a
AST (U/L)	59.74 ± 2.89 c	73.01 ± 4.02 b	79.53 ± 4.29 b	104.3 ± 0.98 a
ALT (U/L)	13.88 ± 0.23 c	19.86 s± 0.81 b	21.96 ± 0.42 b	29.83 ± 2.56 a
ALP (U/L)	9.61 ± 0.31 c	12.63 ± 0.92 b	15.22 ± 0.18 b	21.24 ± 1.53 a

Data represent means ± SEM and means having different letters in the same row are significantly different at *p* < 0.05. AST: Aspartate transaminase, ALT: Alanine transaminase, ALP: Alkaline phosphatase.

## Data Availability

All data sets collected and analyzed during the current study are available from the corresponding author on fair request.

## Supplementary Materials

The following are available online at https://www.mdpi.com/2076-2615/11/3/652/s1, Figure S1: Transmission electron microscopy (TEM) image of CuONPs nanorods with average size of 68.92 ± 3.49 nm., Figure S2: Zeta potential of CuONPs (equal to −15.5 mV), Figure S3: Composition analysis by energy-dispersive X-ray spectroscopy (EDX) of the constituents showing the presence of copper and oxygen elements, Table S1: Results of the 96 h LC50 experiment of copper oxide nanoparticles (CuONPs) in Nile tilapia fingerlings according to Finney’s probit analysis.
