# Supplementary material for: Copper Oxide Nanoparticles Alter Serum Biochemical Indices, Induce Histopathological Alterations, and Modulate Transcription of Cytokines, HSP70, and Oxidative Stress Genes in Oreochromis niloticus
Source: Animals (Basel). 2021 Mar 1;11(3):652. doi: 10.3390/ani11030652 (PMC8001779; doi:10.3390/ani11030652)
Supplement: Supplementary file 1 [file animals-11-00652-s001.pdf]

animals MDPI

Table S1. Results of the 96 h LC_{50} experiment of copper oxide nanoparticles (CuONPs) in Nile tilapia fingerlings according to Finney's probit analysis.

Point	Concentration (mg/L)	95% Confidence Limits Upper	Lower	Slope ± S.E.	Intercept	Chi-test (χ^2) Sig
LC 46.00	90.759	125.335	65.722	2.223 ± 0.072	0.547	0.996
LC 47.00	93.156	128.645	67.457			
LC 48.00	95.611	132.035	69.235			
LC 49.00	98.128	135.511	71.058			
**LC 50.00**	**100.709**	139.076	72.927			
LC 51.00	103.358	142.734	74.845			
LC 52.00	106.079	146.491	76.815			
LC 53.00	108.875	150.352	78.840			
LC54.00	111.750	154.322	80.922			

Control group (theoretical spontaneous response rate) = 0.0000; Bold value indicates the 96 h LC_{50}.

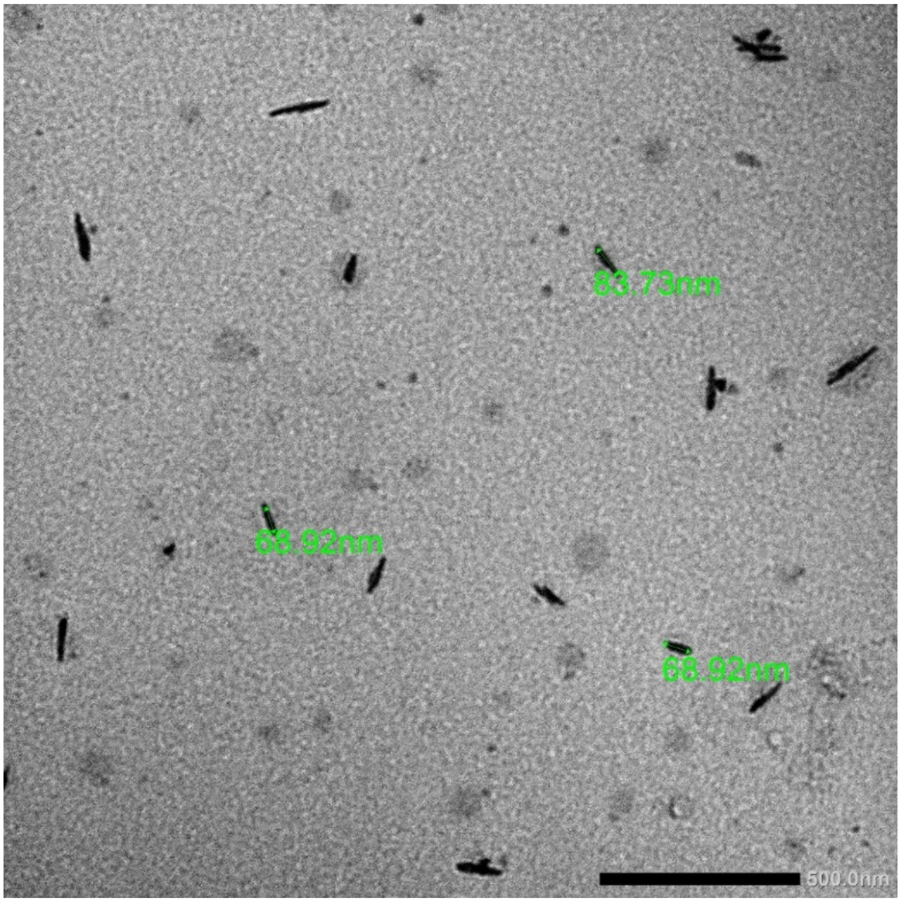

Figure S1. Transmission electron microscopy (TEM) image of CuONPs nanorods with average size of 68.92 ± 3.49 nm.

			Mean (mV)	Area (%)	Width (mV)
Zeta Potential (mV):	-15.5	Peak 1:	-15.5	100.0	6.01
Zeta Deviation (mV):	6.01	Peak 2:	0.00	0.0	0.00
Conductivity (mS/cm):	0.467	Peak 3:	0.00	0.0	0.00

Result quality : Good

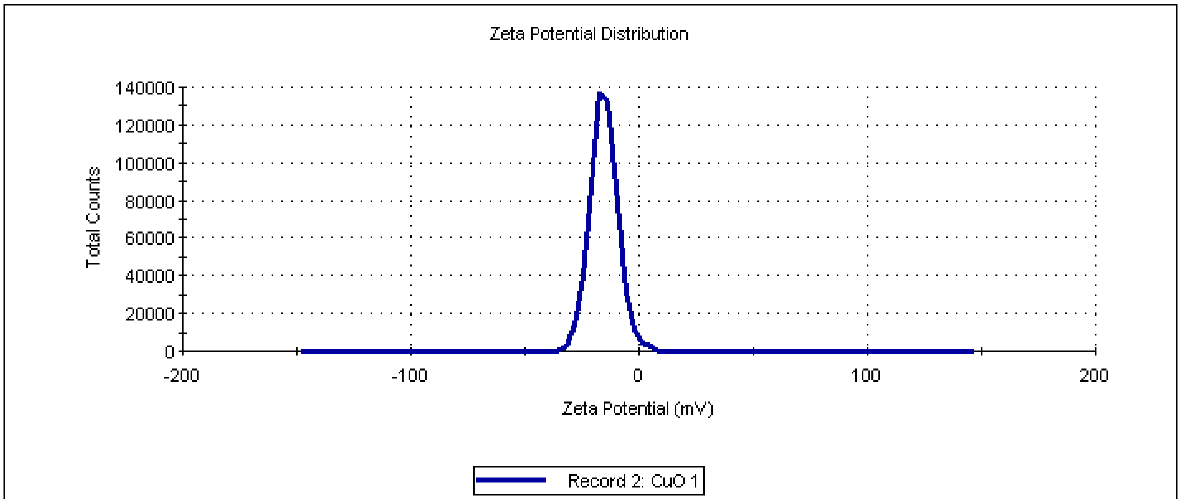

Figure S2. Zeta potential of CuONPs used in the current experiment (equal to -15.5 mV).

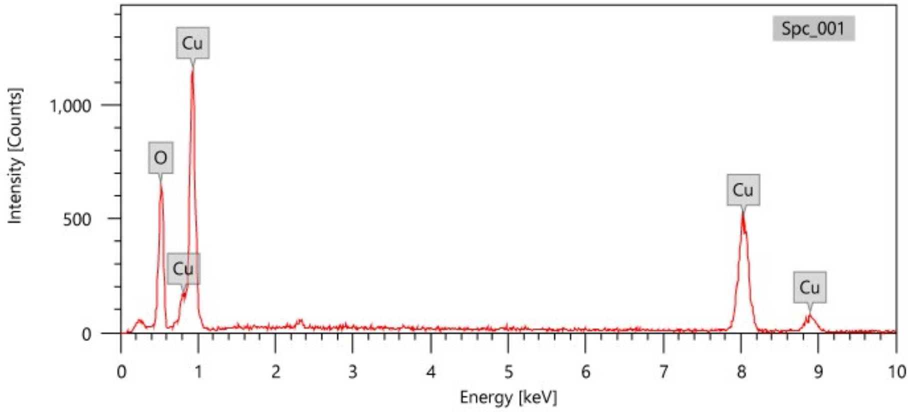

Items	Value
measurement conditions	
Acceleration voltage	20.00 kV
Probe current	-
Magnification	x 80
Process time	T2
Measurement detector	First
Live time	30.00 seconds
Real time	30.11 seconds
Dead time	0.00 %
Count rate	1396.00 CPS

Display name	Standard data	Quantification method	Result Type
Spc_001	Standardless	ZAF	Oxide (Number of oxygen 24)

Chemical formula	Line	Mass%	Mol%	Cations
O	K			
CuO	K	100.00±1.50	100.00±1.50	24.00
Total		100.00	100.00	
Spc_001				Fitting ratio 0.0570

Figure S3. Composition analysis by energy-dispersive X-ray spectroscopy (EDX) of the con-stituents of CuONPs showing the presence of copper and oxygen elements. Characterization of CuONPs used in the present study.
